# Positive associations between triglyceride-glucose (TyG) and TyG-body mass index (TyG-BMI) with cardiovascular disease in patients with psoriasis: cross-sectional results from the SPEECH

**DOI:** 10.3389/fimmu.2025.1644887

**Published:** 2025-09-05

**Authors:** Ying Li, Dawei Huang, Biao Ma, Yuxiong Jiang, Bo Yu, Jianfeng Zheng, Yangfeng Ding, Yuling Shi

**Affiliations:** ^1^ Department of Dermatology, Shanghai Skin Disease Hospital, Tongji University School of Medicine, Shanghai, China; ^2^ Institute of Psoriasis, Tongji University School of Medicine, Shanghai, China

**Keywords:** psoriasis, cardiovascular disease, triglyceride-glucose, TyG-body mass index, insulin resistance

## Abstract

**Background:**

Patients with psoriasis have an increased risk of developing cardiovascular disease(CVD), yet reliable potential markers for cardiovascular risk assessment remain insufficient. The triglyceride-glucose (TyG) index and TyG-body mass index (TyG-BMI) have been identified as potential indicators of metabolic and cardiovascular risk in the general population. However, their specific role in psoriasis-related CVD has not been systematically evaluated.

**Objective:**

This study aimed to investigate the relationship between TyG index, TyG-BMI index and CVD in patients with psoriasis.

**Methods:**

This cross-sectional study utilized data from the Shanghai Psoriasis Efficacy Evaluation Cohort (SPEECH), which includes Chinese patients with moderate to severe psoriasis. Defined by strict inclusion and exclusion criteria, 1112 psoriasis patients were included. CVD included any cerebrovascular events and coronary atherosclerotic heart disease. Multivariate logistic regression and restricted cubic spline analysis (RCS) were used to examine the relationship between TyG index, TyG-BMI index, and CVD risk. Subgroup and sensitivity analyses were conducted to validate the robustness of the findings.

**Results:**

Among the study participants, 223 (20.1%) had CVD. Compared to non-CVD patients, those with CVD were older, had a higher BMI, a higher prevalence of hypertension and diabetes, a longer course of psoriasis, higher levels of blood glucose, triglycerides, and higher levels of TyG index and TyG-BMI index. Subsequent multivariate logistic regression analysis showed a significant positive association between TyG index, TyG-BMI index with CVD risk in psoriasis patients. RCS analysis showed that there was a dose-response relationship between TyG index and TyG-BMI index with CVD risk. Subgroup analysis showed that TyG index and TyG-BMI index were significantly associated with CVD in most subgroups. Patients who were excluded from systematic therapy were subjected to sensitivity analysis, and the results were consistent with the main analysis, suggesting a robust correlation between TyG index and TyG-BMI index and CVD.

**Conclusion:**

Elevated TyG and TyG-BMI indices are independently associated with CVD in psoriasis patients, suggesting their potential as practical markers for cardiovascular risk stratification in this population.

## Introduction

Psoriasis is a chronic inflammatory skin disease affects approximately 2-3% of the global population. Its impact on the body has gone beyond the skin. Existing epidemiologic studies reveal patients with psoriasis have an increased risk for cardiovascular disease (CVD), which are common causes of morbidity and mortality in psoriasis (1–5). The early intervention of cardiovascular abnormalities is therefore of utmost importance for improving the prognosis of these patients. The link between psoriasis and CVD is thought to be related to the systemic inflammation and metabolic disturbances seen in psoriatic patients (1). In recent years, interest has grown in identifying biomarkers that may help evaluate CVD burden in psoriasis patients.

Insulin resistance(IR) is a key driver of both metabolic dysfunction and CVD. Several lipid- and glucose-derived indices have emerged as reliable surrogate markers for insulin resistance (IR), including the triglyceride-glucose (TyG) index, TyG-body mass index(TyG-BMI), Triglycerides to High-Density Lipoprotein Cholesterol ratio (TG/HDL-C), the metabolic score for insulin resistance (METS-IR) and homeostatic model assessment of IR (HOMA-IR) (6, 7). Among them, the TyG index combines two key metabolic parameters, fasting triglycerides (TG) and fasting plasma glucose (FPG), and can sensitively reflect the IR status. It is simple to calculate, cost-effective, and widely accessible (8). Recent study have shown that the combination of TyG index and BMI can significantly enhance the effectiveness of IR (9). Several observational studies have suggested an association between elevated TyG and TyG-BMI indices and an increased risk of CVD in the general population (10, 11). However, the specific impact of these index on CVD risk in patients with psoriasis remains unclear and has not been extensively studied.

Therefore, the aim of this study is to investigate the association between TyG index, TyG-BMI index and CVD in patients with psoriasis. Understanding the association between these index and CVD risk in psoriasis patients may provide insights into potential biomarkers for identifying those at increased risk of cardiovascular complications.

## Methods

### Study design

This cross-sectional study is based on data from the Shanghai Psoriasis Effectiveness Evaluation Cohort (SPEECH). SPEECH is a multicenter observational cohort study of psoriasis in China, which includes psoriasis patients treated with acitretin, methotrexate, phototherapy, and various biologics. The aim of SPEECH is to investigate the characteristics of psoriasis in the Chinese population and to identify an appropriate treatment protocol for psoriasis that takes into account the specific needs of the Chinese population and national conditions. The Ethics Committee of the Shanghai Skin Disease Hospital (#2020-36) thoroughly reviewed this prospective cohort study. All participants provided informed consent.

### Participants

Patients were screened from the SPEECH database for inclusion in this study. The inclusion criteria are as follows: 1. Adults diagnosed with moderate-to-severe plaque psoriasis; 2. Routine blood tests, fasting glucose, and triglyceride tests require venous blood samples to be collected early in the morning. The main exclusion criteria include a lack of baseline information, venous blood samples or other important data.

### Data acquisition

All analysis data were directly extracted from the database and included variables such as age, gender, Body Mass Index(BMI), smoking history, drinking history, hypertension history, diabetes history, duration of psoriasis, psoriatic arthritis (PSA), psoriasis area and severity index (PASI), and family history of psoriasis. In addition, blood samples were collected from all participants in the early morning to analyze fasting blood glucose and triglyceride levels. All clinical and laboratory data were collected at the time of cohort entry (baseline), prior to initiation or change of systemic therapy, as part of the standardized SPEECH baseline assessment. Smoking and drinking were categorized as “Never” or “Past or current”. Psoriasis-related inquiries, assessment of PASI, and diagnosis of PSA were independently performed by a specialized dermatologist. The diagnosis of PSA was based on the CASPAR diagnostic criteria. Comorbidity data for psoriasis were obtained through self-reported information on a health questionnaire or from past medical records. Participants were asked, “Have you ever been diagnosed with hypertension/diabetes/MAFLD by a doctor?” A response of “yes” indicated a case of hypertension/diabetes/MAFLD. If the patient had a clear diagnosis of hypertension, diabetes, or MAFLD in their medical record, it was considered as a case of hypertension, diabetes, or MAFLD.

Data on CVD were also obtained through questionnaires and past medical records. Participants were asked, “Has a doctor or other healthcare professional ever told you that you have congestive heart failure/coronary heart disease/angina pectoris/myocardial infarction/stroke?” Any response of “yes” to these questions indicated a case of CVD. If the patient had a clear diagnosis of any of these diseases in their previous medical record, it was considered as a case of CVD. TyG is defined as Ln [triglyceride (mg/dL)×fasting blood glucose (mg/dL)/2]; and TyG-BMI is calculated as TyG x BMI (kg/m2).

### Statistical analysis

The primary method of analysis in this study is cross-sectional analysis. The group was divided into two categories based on the presence or absence of CVD. Continuous variables that exhibit a normal distribution are reported as mean ± standard deviation (SD) and evaluated using the student t-test. Non-normally distributed continuous variables were presented with the median (interquartile range [IQR]) and compared using the Mann-Whitney U test. Categorical variables are expressed as frequency (%) and evaluated using Chi-square tests or Fisher’s exact tests.

The patients were divided into three groups according to the tertiles level of the TyG and TyG-BMI in ascending order of Q1, Q2, and Q3. Covariates included age, gender, BMI, smoking history, drinking history, hypertension, diabetes, PSA, duration of psoriasis, family history and PASI score based on previous literature. Three models were developed to investigate the correlation: Model 1 did not include any covariates, Model 2 only adjusted for age, gender, smoking history, drinking history, and BMI, and Model 3 included all covariates for analysis. When using TyG-BMI as the target variable, BMI was not used as a covariate. Odds ratios (OR) and 95% confidence intervals (95% CI) for different groups of IR indictors were calculated using univariate logistic regression and multivariate logistic regression. And the trend test was also carried out. Multiple linear regression describes the relationship between the IR indicator and CVD when TyG and TyG-BMI indices considered as a numerical variable. Subgroup analysis was then conducted to further explore potential interactions and influencing factors. Due to the potential impact of psoriasis systemic treatment on CVD, patients who had previously received systemic therapy were excluded from the sensitivity analysis. A p-value < 0.05 was considered statistically significant. All statistical analyses were performed using R version 4.2.1.

## Results

### Characteristics of the study participants

**Figure 1:**
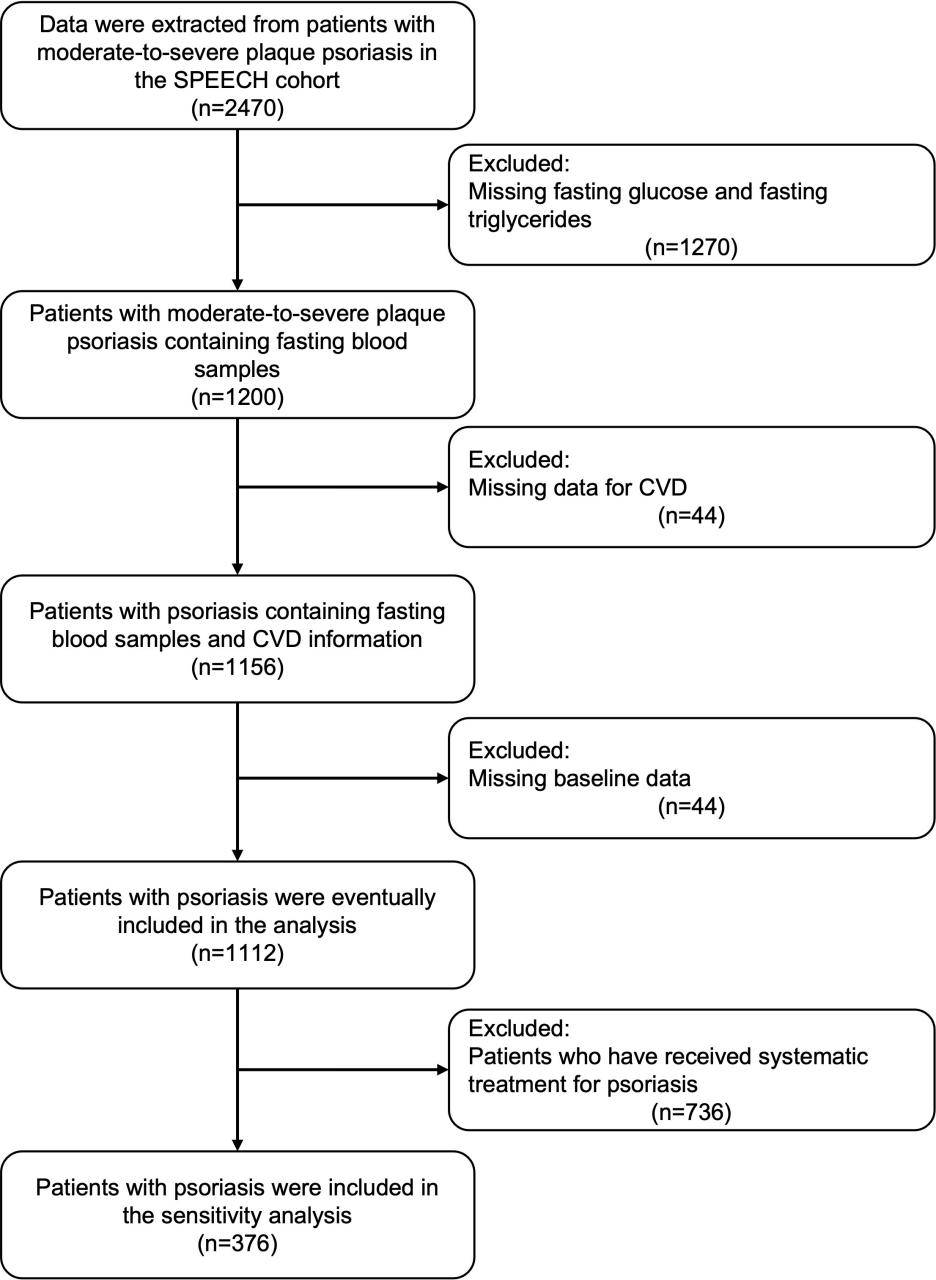
Flow chart of the study.

After screening the patient information of the SPEECH cohort according to the exclusion criteria, a total of 1112 patients were included in the present study (**Figure 1**). Among them, 223 patients (20.1%) were found to have CVD. Consistent with previous studies, patients with CVD had a higher BMI, higher prevalence of hypertension and Type 2 diabetes mellitus (T2DM), and higher levels of fasting blood glucose, triglycerides, TyG, and TyG-BMI. Surprisingly, patients with CVD had a shorter disease duration and milder PASI (**Table 1**).

**Table 1:** Demographic and clinical characteristics of all patients with psoriasis.

Characteristic	Overall (N = 1112)	Non-CVD (N = 889)	CVD (N = 223)	P value
General characteristics				
Age (year), Median (IQR)	58 (42, 66)	55 (39, 64)	65 (59, 72)	<0.001
Female	253 (22.8%)	211 (23.7%)	42 (18.8%)	0.119
Smoking-Past or current	329 (29.6%)	270 (30.4%)	59 (26.5%)	0.252
Drinking-Past or current	177 (15.9%)	143 (16.1%)	34 (15.2%)	0.759
BMI (kg/m2), Median (IQR)	24.2 (22.1, 26.9)	24.2 (21.9, 26.9)	24.6 (22.6, 27.0)	0.041
Comorbidities				
Hypertension	367 (33.0%)	243 (27.3%)	124 (55.6%)	<0.001
T2DM	212 (19.1%)	142 (16.0%)	70 (31.4%)	<0.001
MAFLD	422 (37.9%)	328 (36.9%)	94 (42.2%)	0.148
Evaluation of psoriasis				
PsA	231 (20.8%)	191 (21.5%)	40 (17.9%)	0.243
Duration (year), Median (IQR)	10 (6, 20)	10 (6, 20)	15 (8, 23)	0.002
PASI, Median (IQR)	15 (12, 21)	16 (12, 21)	15 (10, 20)	0.018
Family history of psoriasis	178 (16.0%)	146 (16.4%)	32 (14.3%)	0.450
Laboratory examination				
Fasting blood glucose (mg/dL), Median (IQR)	93 (84, 108)	91 (84, 103)	99 (86, 125)	<0.001
Triglyceride (mg/dL), Median (IQR)	125 (93, 172)	122 (91, 169)	136 (103, 183)	0.003
TyG, Median (IQR)	9.30 (8.90, 9.70)	9.20 (8.90, 9.70)	9.50 (9.10, 9.80)	<0.001
TyG-BMI, Median (IQR)	225 (203, 255)	223 (201, 253)	235 (214, 263)	<0.001

IQR, interquartile range; BMI, body mass index; T2DM, Type 2 diabetes mellitus; MAFLD, metabolic-associated fatty liver disease; PSA, psoriatic arthritis; PASI, psoriasis area and severity index; TyG, triglyceride-glucose; TyG-BMI, triglyceride glucose-body mass index.

### Association between TyG index and CVD in psoriasis

The relationships between TyG-related index and CVD are presented in **Table 2**. We found that TyG and TyG-BMI were significantly associated with CVD in psoriasis, with a dose-dependent correlation observed in Q2 and Q3 compared to Q1.

**Table 2:** Association of the TyG index, TyG-BMI index with cardiovascular disease in patients with psoriasis.

Models	Each 1-unit increase	Q1	Q2	Q3	P-trend
TyG					
Model 1	2.161 (1.709-2.750)	Ref.	1.970 (1.287-3.069)	2.791 (1.866-4.264)	<0.001
Model 2	2.643 (2.018-3.492)	Ref.	1.891 (1.195-3.040)	2.959 (1.901-4.701)	<0.001
Model 3	2.388 (1.791-3.211)	Ref.	1.854 (1.157-3.018)	2.537 (1.586-4.134)	<0.001
TyG-BMI					
Model 1	1.007 (1.003-1.010)	Ref.	1.869 (1.285-2.742)	1.775 (1.216-2.613)	0.004
Model 2	1.013 (1.008-1.017)	Ref.	1.925 (1.289-2.899)	2.525 (1.667-3.865)	<0.001
Model 3	1.010 (1.006-1.015)	Ref.	1.703 (1.125-2.599)	1.936 (1.232-3.067)	0.005

Ref, reference.

Model 1: unadjusted.

Model 2: analyzed adjusted for age, gender, smoking, drinking, and BMI.

Model 3: adjusted for all covariates.

**Figure 2:**
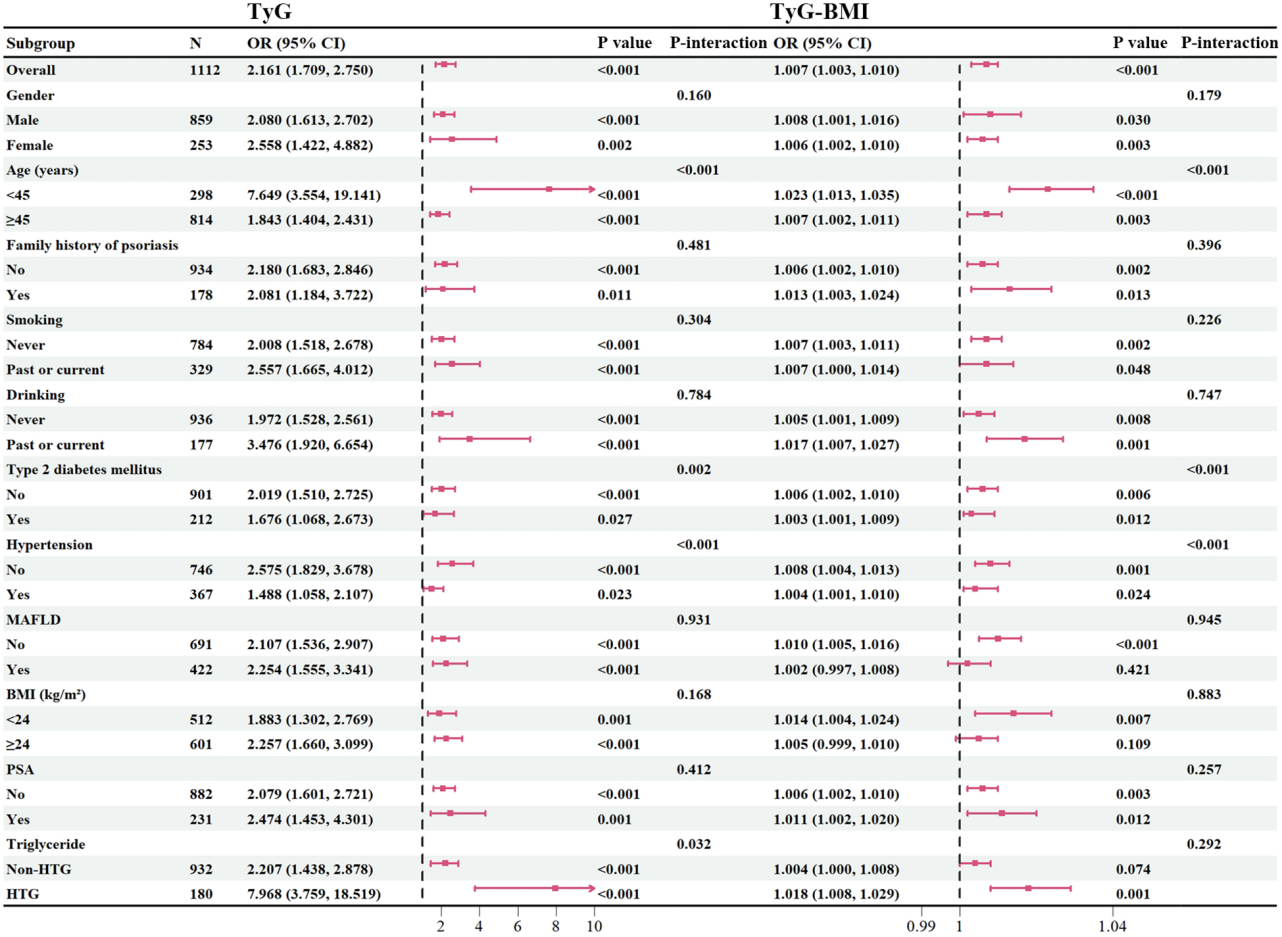
Subgroup analysis. BMI, body mass index; PSA, psoriatic arthritis; TyG, triglyceride-glucose; TyG-BMI, triglyceride glucose-body mass index; HTG, hypertriglyceridemia.

To investigate the presence of subgroup differences in terms of the association between continuous TyG-related index and CVD, we conducted subgroup analyses according to the potential atherosclerosis risk factors, including age, gender, family history of psoriasis, smoking/drinking history, BMI, and comorbidities (**Figure 2**). Overall, there were no significant interactions detected in most categories (*P* > 0.05), except age, hypertension, T2DM for the outcomes of CVD. However, these variables appear to have minimal impact on the robustness of the results, as TyG-related index remain significantly associated with CVD in psoriasis across all subgroups (*P* < 0.05). It is worth noting that in patients with elevated triglycerides, the association between TyG and CVD was still maintained a good correlation.

### Sensitivity analysis

A sensitivity analysis was performed excluding patients who had previously received systemic treatments that might influence cardiovascular risk profiles. Acitretin and Tumor Necrosis Factor-α(TNF-α) inhibitors were selected because of their potential metabolic and inflammatory effects that could confound associations between TyG-related indices and CVD. Although acitretin is not typically associated with an increased risk of major cardiovascular events, it has been linked to alterations in lipid metabolism, which might affect TyG index values. TNF-α inhibitors may improve cardiovascular outcomes via systemic inflammation reduction, potentially attenuating observed associations. We further assessed the predictive ability of TyG-related index for CVD beyond established risk factors in basic Models 1, 2 and 3. We found that TyG were significantly associated with CVD in psoriasis, with a dose-dependent correlation observed in Q2 and Q3 compared to Q1 (**Table 3**) in Model 1 to 3. But TyG-BMI was only associated with CVD in psoriasis in Model 2 in sensitivity analysis.

**Table 3:** Results of sensitivity analysis(N=376).

Models	OR	Q1	Q2	Q3	P-trend
TyG					
Model 1	2.463 (1.689-3.677)	Ref.	3.512 (1.599-8.575)	4.632 (2.181-11.064)	<0.001
Model 2	3.779 (2.337-6.379)	Ref.	3.934 (1.707-10.023)	5.893 (2.561-15.110)	<0.001
Model 3	3.362 (2.063-5.745)	Ref.	3.524 (1.505-9.092)	4.849 (2.060-12.657)	0.001
TyG-BMI					
Model 1	1.009 (1.003-1.015)	Ref.	1.914 (1.039-3.617)	1.772 (0.940-3.409)	0.091
Model 2	1.013 (1.006-1.021)	Ref.	1.956 (1.020-3.847)	2.472 (1.245-5.052)	0.011
Model 3	1.012 (1.004-1.020)	Ref.	1.825 (0.924-3.685)	1.973 (0.933-4.255)	0.082

Ref, reference.

Model 1: unadjusted.

Model 2: analyzed adjusted for age, gender, smoking, drinking, and BMI.

Model 3: adjusted for all covariates.

## Discussion

Psoriasis has been increasingly characterized as a systemic inflammatory disorder that contributes to heightened cardiovascular disease (CVD) risk. Identifying potential biomarkers of CVD risk in psoriasis patients is therefore critical to facilitate early intervention and improve long-term outcomes. In the present study, we found that an increase in TyG related-index was significantly associated with CVD in psoriasis patients. These associations persisted across various subgroups and remained robust in sensitivity analyses. Our finding demonstrated that TyG related-index is associated with the occurrence of CVD in psoriasis and can be used as a simple and effective cardiovascular risk assessment tool in daily practice.

A convincing deal of evidence supports that psoriasis is associated with CVD (12–14). A prospective, population-based cohort study of 130,000 patients with psoriasis and 500,000 controls reported an overall 50% increased risk of myocardial infarction(MI) in patients with psoriasis (15). Compared to controls, severe psoriasis confers the highest CV risk, including up to a 3-fold increased odds of MI, 60% higher odds of stroke, and 40% higher odds of CV death (15). A meta-analysis including 75 studies with 503,686 psoriasis patients reported up to a 50% increased odds of CVD in psoriasis compared to controls without psoriasis (16). However, some studies have shown no correlation between psoriasis and CVD risk (17–19). The conflicting results may be related to region and race, disease severity, disease course, follow-up time, confounding factors, etc.

The precise underlying mechanisms linking psoriasis and CVD are not well defined. Psoriasis shares common pathophysiologic mechanisms with atherosclerosis and cardiovascular risk factors. The Polymorphism of interleukin (IL)-23R and IL-23 genes, as well as other genes involved in lipid and fatty-acid metabolism, renin-angiotensin system and endothelial function, have been described in patients with psoriasis and with cardiovascular risk factors. Moreover, systemic inflammation in patients with psoriasis, including elevated serum proinflammatory cytokines (e.g.,TNF-α, IL-17, and IL-23) may contribute to an increased risk of atherosclerosis, hypertension, alteration of serum lipid composition, and IR (20). Platelets overactivation may contribute to initiation and progression of immune responses in lesional skin and blood vessels, ultimately leading to psoriasis and its comorbidities such as atherosclerosis, ischemic heart disease, stroke, MI, and other CVD (21). An clinical randomized trial (RCT) indicates that psoriatic patients treated with TNF inhibitors have a significantly lower risk of MI (22).

The TyG index is a surrogate marker of IR, a central feature of metabolic syndrome. The prevalence of metabolic syndrome is increased in psoriasis and has been linked to higher coronary atherosclerosis in this population (23, 24). The TyG index relates to cardiometabolic risk factors, IR, and subclinical atherosclerosis in psoriasis (25). The link between psoriasis and metabolic syndrome is partly attributed to systemic inflammation. However, other chronic inflammatory skin diseases such as atopic dermatitis also involve systemic inflammation, but the association with metabolic syndrome is less clear (26). Differences in immune activation may contribute to this discrepancy. Nonetheless, CVD has also been reported in atopic dermatitis, even in subclinical stages (27, 28). Several studies have identified a link between hidradenitis suppurativa (HS) and CVD (29–31). Therefore, the TyG index also has a potential role in detecting CVD in other chronic inflammations such as atopic dermatitis and HS. Although the metabolic participation in these chronic inflammations is relatively small, the cardiovascular risk is relatively high.

In addition to TyG index, some other indicators such as the ratio of neutrophils to lymphocytes (NLR) and the ratio of monocytes to high-density lipoprotein (MHR) are also used to assess cardiovascular risk factors (32, 33). These indicators integrate inflammation and lipid metabolism, making up for the deficiencies of traditional risk assessment in patients with psoriasis, and is low-cost and easily accessible. With growing recognition of the systemic implications of psoriatic disease, dermatologists are increasingly engaged in cardiovascular risk management (34, 35) Incorporating simple and reliable markers like TyG, NLR, or MHR into dermatologic practice may help identify high-risk patients earlier, guide timely referrals, and improve long-term health outcomes.

One of the major strengths of this study is the use of a large, well-characterized psoriasis cohort, allowing for a comprehensive evaluation of TyG index and TyG-BMI index in relation to CVD. Additionally, the robust statistical approach, including multivariate adjustments, subgroup analyses, and sensitivity analyses, ensures the reliability of our findings. From a clinical perspective, our study highlights the need to integrate metabolic markers such as TyG index and TyG-BMI index into routine cardiovascular risk assessment for psoriasis patients. Unlike traditional markers, these indices are easily calculated from fasting blood glucose and triglyceride levels, making them accessible and cost-effective for widespread clinical application.

This study has several limitations. First, given the cross-sectional data structure, causation cannot be established regarding the association. The further accumulation of cases with a prospective cohort study are needed to prove that causal link. Second, although we adjusted for multiple confounders, residual confounding cannot be completely excluded due to the observational nature of our analysis. Third, our study cohort was derived from a single ethnic population, which may limit the generalizability of our findings to other populations. Lastly, although we have demonstrated the association among TyG index, TyG-BMI index and CVD, the role of low-density lipoprotein cholesterol (LDL-C) as a traditional cardiovascular risk factor in patients with psoriasis still cannot be ignored. There is a potential synergistic effect between TyG index and LDL-C in the risk of cardiovascular onset in psoriasis. Therefore, in the CVD risk assessment of patients with psoriasis, the combined detection of TyG index and LDL-C can provide more comprehensive risk stratification information. Future studies can further explore the interaction between TyG index and LDL-C to optimize the cardiovascular risk management strategies for patients with psoriasis.

## Conclusion

In the psoriasis population, an elevated level of TyG index and TyG-BMI index is linked to cardiovascular diseases, even after adjusting for traditional cardiovascular diseases risk factors. With the advantages of being simple, accessible and reliable, the TyG index and TyG-BMI index may be a useful tool for cardiovascular risk assessment for psoriasis patients in daily practice.

## Data Availability

The original contributions presented in the study are included in the article/supplementary material. Further inquiries can be directed to the corresponding author/s.
